# Mitochondrial Involvement in Cisplatin Resistance

**DOI:** 10.3390/ijms20143384

**Published:** 2019-07-10

**Authors:** Veronica Cocetta, Eugenio Ragazzi, Monica Montopoli

**Affiliations:** 1 Department of Pharmaceutical and Pharmacological Sciences, University of Padova, Largo Egidio Meneghetti 2, 35131 Padua, Italy; 2 Venetian Institute of Molecular Medicine (VIMM), Via Orus 2, 35129 Padua, Italy

**Keywords:** mitochondria, cisplatin, resistance, mitochondrial dynamics, mtDNA

## Abstract

Cisplatin is one of the worldwide anticancer drugs and, despite its toxicity and frequent recurrence of resistance phenomena, it still remains the only therapeutic option for several tumors. Circumventing cisplatin resistance remains, therefore, a major goal for clinical therapy and represents a challenge for scientific research. Recent studies have brought to light the fundamental role of mitochondria in onset, progression, and metastasis of cancer, as well as its importance in the resistance to chemotherapy. The aim of this review is to give an overview of the current knowledge about the implication of mitochondria in cisplatin resistance and on the recent development in this research field. Recent studies have highlighted the role of mitochondrial DNA alterations in onset of resistance phenomena, being related both to redox balance alterations and to signal crosstalk with the nucleus, allowing a rewiring of cell metabolism. Moreover, an important role of the mitochondrial dynamics in the adaptation mechanism of cancer cells to challenging environment has been revealed. Giving bioenergetic plasticity to tumor cells, mitochondria allow cells to evade death pathways in stressful conditions, including chemotherapy. So far, even if the central role of mitochondria is recognized, little is known about the specific mechanisms implicated in the resistance. Nevertheless, mitochondria appear to be promising pharmacological targets for overcoming cisplatin resistance, but further studies are necessary.

## 1. Introduction

*Cis*-diamine-dichloroplatinum (II) (best known as cisplatin or CDDP) is the most employed platinum-based compound and the first approved by FDA (Food and Drug Administration) in 1978 for the treatment of testicular and bladder cancer. It exerts clinical activity in the treatment of a wide variety of cancers, including ovarian, testicular, bladder, head and neck, cervical, lung and colorectal cancers [1,2,3] either alone or in combination with other anticancer agents like paclitaxel, doxorubicin, 5-FU etc. [4].

Despite toxicity even at low doses (especially nephrotoxicity and ototoxicity), cisplatin remains the first-line therapy for several types of solid tumors [5]. 

In CDDP-based therapy, the treatment often is effective in inducing an initial therapeutic success associated with a cancer stabilization or partial response. Nevertheless, many patients present an intrinsic resistance to the drug or develop cisplatin resistance during the course of the treatment, leading to therapy failure [6,7]. 

In particular, colorectal, prostate and lung cancers are intrinsically resistant to cisplatin while acquired resistance is more often observed in ovarian cancer patients [8,9,10]. The clinically acquired resistance appears to be a multifactorial phenomenon, which involves several unrelated mechanisms exploited simultaneously within the same cell and since now, little is known about this intricate process. Researches highlight how the acquisition of resistance can be due to decrease in drug accumulation, which includes reduced uptake or increased efflux of the drugs, enhanced drug detoxification system due to high levels of scavengers such as glutathione/metallothioneins, increased tolerance to damaged DNA, increased DNA repair mechanisms or to a metabolic rewiring of the cells in order to elude cisplatin-induced death [11,12,13,14,15]. These mechanisms are peculiar to each cancer cell line in such a way that a particular tumor may exhibit one, two or even all the above-mentioned resistance mechanisms [16].

Cisplatin can enter the cells by passive diffusion but recently a lot of interest has been given to active uptake of the drug. In particular, for the transport of cisplatin into the cells via facilitated diffusion, the copper transporter proteins (CTR1 and CTR2) seem to be involved in the uptake of the drug [17,18]. Once inside the cell, the activation of CDDP is due to the different chloride concentration between blood plasma (~100 mM) and cell cytoplasm (~4 mM). This drop in the chloride concentration facilitates the mono- or diaquation of the drug making it a potent electrophile prone to react with a variety of nucleophilic sites, especially nucleic acids and sulfhydryl groups of proteins. For these reasons, DNA is thought to be the primary target of cisplatin [4,11]. The platinum atom of CDDP forms covalent bonds with the purine bases (with the N7-position) producing 1,2- or 1,3-intrastrand crosslinks and a lower percentage of interstrand crosslinks. This formation of CDDP-DNA adducts interferes with DNA replication and transcription, and the formation of crosslinks disrupts the structure of DNA which is recognized by cellular proteins to repair the DNA damage [19]. Studies of the last decade have shown that only a very tiny percentage of CDDP (1%) interacts with nuclear DNA, while the largest amount interacts with sulfur donors (such as cysteines, methionines, thiols, etc.), proteins or mitochondrial structures, as well as with the mitochondrial DNA (mtDNA) [12,20].

The study of the details of the binding of platinum compounds to nucleic acids and to other cellular structures has not only led to a better understanding of the mechanism of action, but may also result in the development of new drugs, new formulations or of special drug-dosing protocols, making use of combined therapies with protective —rescue or synergic—agents [21,22]. Moreover, it is important to underline that to comprehend the overall pharmacological and toxicological profile of platinum drugs, it is necessary to explore alternative intracellular pathways and interactions. The identification of different pathways and molecular targets may offer new perspective for overcoming resistance phenomena and to reduce the toxic effect of the platinum drugs [23]. 

## 2. Mitochondria

Mitochondria are dynamic subcellular organelles which are capable of rapidly sensing stress signals, thus coordinating several biochemical pathways required to adapt to environmental changes. 

A hypothesis about the origin of mitochondria suggests that several billion years ago, a precursor of modern eukaryotic cell swallowed an α-proteobacterium giving rise to the mitochondrion [24]. In the last few years, this endosymbiotic origin was confirmed by proteomics, genomics and bioinformatics techniques [25,26].

While originally relegated to the role of “energy powerhouse of the cells”, it is now well established that mitochondria are the hub of numerous signal pathways implicated in most cellular processes. In fact, besides exerting central bioenergetics functions via tricarboxylic acid cycle (TCA), oxidative phosphorylation (OXPHOS) and fatty acid oxidation (FAO), mitochondria exert also anabolic functions like biosynthesis of amino acids, lipids, and nucleotides. Moreover, they provide maintenance of homeostatic levels of Ca^2+^ and of reducing equivalent carriers and are involved in the intrinsic apoptotic signaling pathway hence governing cell death [27,28].

These various functions of mitochondria enable them to sense cellular stress and allow them to confer a high level of plasticity to cells, which permits a fast adaptation to challenge microenvironmental conditions. 

It is, however, evident, and firmly confirmed the fundamental role of mitochondria metabolism on all steps of oncogenesis, from malignant transformation to tumor progression and response to treatment [29]. 

## 3. Mitochondria and Cancer

The first description of the role of mitochondria in cancer metabolism is linked to Warburg. He was the first to demonstrate that tumor cells present an altered energy metabolism relying more on glycolysis with respect to oxidative phosphorylation, even in presence of high oxygen tension. Warburg hypothesized that mitochondrial dysfunction causes the necessity of cancer cells to rely on glycolysis as the only alternative for ATP production [30]. Today, this “Warburg effect” is currently referred to as “metabolic reprogramming” and is a recognized hallmark of cancer [31,32] even if the spotlight of metabolic transformation research is today focused on the anabolic processes. This oxidative phosphorylation provides a biosynthetic advantage to tumor cells, diverting energy substrate into reactions which lead to the production of building blocks for high-proliferative cancer cells (amino acids, lipids, and nucleotides). Besides producing ATP, the oxidative phosphorylation is the major source of ROS within the mitochondrion and the entire cell. The most notable sites of ROS production are complexes I, II and III of the mitochondrial electron transport chain. The early observation that cancer cells present high ROS levels, led to the idea that ROS inhibition could be a useful therapeutic strategy [33,34,35]. However, in the past two decades, a more complex picture has emerged, that depicts the role of ROS in different signaling pathways involved in the control of several physiological and pathological cell processes [36,37]. In response to elevated ROS, many tumors present an upregulation of protective antioxidant pathways able to neutralize ROS. Superoxide dismutase (SOD2), glutathione, thioredoxin, and peroxiredoxins represent the major antioxidant mechanism exploited by cells in order to maintain redox homeostasis. Cells resistant to chemotherapeutic drugs have been shown to present elevated levels of antioxidant factors able to neutralize the increased ROS production caused by the drug [38,39,40,41]. The upregulation of these antioxidants in cancer can prevent ROS-mediated cytotoxicity and may give a selective advantage in tumor cells growing [35]. Several studies demonstrated that cisplatin-induced cytotoxicity is closely related to ROS generation [42,43]. Increased ROS generation alters mitochondrial membrane potential and induces damages in the respiratory chain triggering to apoptosis. The high presence of antioxidant actors may be one of the mechanisms involved in cisplatin resistance onset and it has been demonstrated that an increase in mitochondrial ROS scavenging reduces cellular sensitivity to cisplatin [44]. Mitochondria are also involved in sequestration and release of Ca^2+^. Calcium signaling between ER-mitochondria and cytosol appears to be a major contributor to the cytotoxic effects of chemotherapy and many chemotherapeutic drugs trigger a rapid onset of cytosolic calcium [45,46,47]. Several studied have already demonstrated that many chemotherapeutic drugs act via Ca^2+^ signaling altering ER-mitochondria calcium transfer [48,49].

Over the years, some interest has been given to proteasome inhibitors in treating refractory cancers; in addition to the cytosolic ubiquitin/proteasome and protein quality control systems, mammalian cells displayed of other families of ATP-dependent proteases located within the mitochondria including ClpXP [50]. A study by Zhang and Maurizi (2016) highlighted an involvement of the mitochondrial ATP-dependentClp matrix protease (ClpP) in cisplatin resistance in human cells. In this in vitro model, ClpP and ClpX (respectively a homolog of the self-compartmentalized protease, ClpP and a homolog of the *Escherichia coli* ATP-dependent protein unfoldase, ClpX), are imported into the mitochondrial matrix where they interact to form the ATP-dependent protease ClpXP and play a role in the mitochondrial unfolded protein response. They found that a reduction of mitochondrial ClpP or ClpX subunits induces a sensitization to cisplatin treatment. HClpXP (human ClpXP) activity positively affects the ability of cells to efflux cisplatin and suggests that targeting these proteins could be a new strategy to sensitize cancer cells to cisplatin [51].

## 4. Mito-Nuclear Crosstalk

Communication among cytoplasm, nucleus and mitochondria is fundamental for maintaining mitochondrial function and cellular homeostasis [52,53]. Bi-directional communication between mitochondria and nucleus has been shown to provide the equilibrium of cellular homeostasis. The anterograde signaling consists in the nuclear control of the mitochondria by regulation of nuclear-encoded mitochondrial genes, while the complementary retrograde signaling is aimed to permit mitochondrial communication with the nucleus [54,55].

The nucleus, in response to environmental signals, directly controls the transcription and translation of genes, regulating mitochondrial biogenesis and OXPHOS functions, via anterograde pathway. On the other site, retrograde signaling is a mitochondrial quality control mechanism by which dysfunctional mitochondria are able to interact with the nucleus, communicating metabolic, oxidative and respiratory stressful conditions [56,57,58]. These retrograde signals activate diverse nuclear responses, promoting multiple pathways that regulate energy homeostasis, oxidative stress, mitophagy, among other functions in other to prime cellular-adaptation strategies. The mitochondria to nucleus crosstalk influences many cellular and cancer phenotypes, including alterations in survival rate, metastasis, metabolism and drug resistance; indeed, it could be a likely mechanism by which altered mitochondrial function modulates adaptive changes in nuclear gene expression and thus in metabolism. The retrograde signaling, besides inducing adaptation in metabolism, can also provide a positive selection for specific mitochondrial DNA mutations inducing a phenotype resistant to chemotherapy [59]. In fact, the signals from damaged mitochondria are considered a homeostatic stress response against intrinsic or extrinsic (for instance chemicals, and toxins) stimuli. 

So far, little is known about the identification of the molecules that can induce a retrograde signaling; since now, several studies have demonstrated how NAD^+^/NADH ratio, acetyl-CoA, ATP, oncometabolites, ROS and Ca^2+^ could be involved in this crosstalk with the nuclear genome [60]. Khurshed et al. (2018) demonstrated that treatment with cisplatin induces a significant mitonuclear protein imbalance in IDH1^MUT^ HCT116 cells which is not obtained by treatment with carboplatin. The mitonuclear protein imbalance was accompanied by a decrease in cellular respiration of cells, indicative of impaired mitochondrial activity [61]. A study by Wang et al. (2016) provided evidence to suggest that the ROS-activated GCN2-Eif2α-ATF4-Xct pathway is retrograde signaling that contributes to mitochondrial dysfunction, enhancing cisplatin resistance in different human gastric cancer cell lines [62]. The involvement of mitochondrial signals to the nucleus in resistance phenomena has not been deeply explored yet. The recent findings in the field indicate that the signals from damaged mitochondria to the nucleus lead to altered expression of nuclear-encoded genes, and thus to a possible reprogram of the metabolism allowing cell adaptation. Further studies will improve the scientific knowledge about this fine-regulated mechanism possibly opening up new perspective in chemotherapy and drug resistance.

## 5. Intercellular Mitochondrial Transfer 

Studies of the recent years have revealed that, besides to be maternally inherited, mitochondria can be horizontally transferred between cells. The transfer permits the incorporation of mitochondria/fragment of them, and mitochondrial DNA in recipient cells, allowing changes in functional properties, bioenergetic profile, and mitochondrial functionality. Spees at al. in 2006 were the first to demonstrate the transfer of functional mitochondria from human stem cells to recipient mitochondria-deprived cells, which led to a recovery in mitochondrial respiration [63]. 

The transfer does not occur by passive uptake, but it is an active process that involves formation of vesicles, exosomes or tunneling nanotubules (TNTs) between cells. Several studied already demonstrated the transfer of mitochondria in different cells models using stem cells or immortalized cells as mitochondria donor. Moreover, the incorporation of exogenous mitochondria into cells has been shown to contribute to alterations in bioenergetic profile not only in vitro but also in vivo [64,65,66,67,68,69]. Evidence supports the idea that the transfer of mitochondria or their components may be involved, besides in the rescue of mitochondrial defects, also in initiation of stem cells differentiation, activation of inflammatory pathways and also in efficient metabolic adaptation of tumor cells to a challenging microenvironment [70]. Therefore, in support of these observations, the possible role of this process in chemoresistance phenomena has been studied. Pasquier et al. have shown that a mitochondrial transfer via TNTs from endothelial cancer cells enhances their chemoresistance to doxorubicin; moreover, they demonstrated that human endothelial and MSCs cells performed a transfer of mitochondria to MDA-MB-231 triple-negative human breast carcinoma cells, and SKOV3 and OVCAR3 ovarian carcinoma cell lines [71]. Interestingly, mitochondria-acceptor cells presented resistance to chemotherapy, underlining a functional aspect of mitochondrial acquisition beyond respiration recovery. Moschoi et al. (2016) firstly demonstrated that in vitro exposure to chemotherapy selects a subpopulation of leukemic cells engaged in physical contact with stromal cells. The contact allows the uptake of up to 16 intact mitochondria by leukemic cells, representing 14% of their total mitochondrial mass. They observed that recipient cells can maintain their mitochondrial transmembrane potential under chemotherapy and are more resistant to the treatment. Moreover, they also demonstrated an in vivo transfer from bone marrow mesenchymal cells to myelogenous leukemia cells which confers chemoresistance to immunodeficient NSG mice [72]. Recently, Boukelmoune et al. (2018) have demonstrated a transfer of mitochondria from mesenchymal stem cells (MSCs) to cisplatin-damaged-neuronal stem cells (NSCs). Their data showed that MSCs can transfer mitochondria to damaged NSCs via formation of tubular structures, thus favoring the latter one survival after cisplatin treatment. The inhibition of actin polymerization in MSCs blocks the transfer of mitochondria eliminating the beneficial effect of MSCs on survival of NSCs. On the contrary, the enhancement of mitochondrial transfer by overexpression of Miro1 protein, a mitochondrial motor protein, further increases the survival of NSCs after cisplatin treatment [73]. 

Therefore, even if little is known about the physiological relevance of this phenomenon and its possible link with chemoresistance, further studies will in the future shed some light on the possible role of mitochondria in transferring chemoresistant-phenotypes to tumor cells.

## 6. Mitochondrial DNA as a Target for Cisplatin

In addition to regulating bioenergetics metabolism, mitochondria represent a central component which integrates epigenetics, stemness, differentiation, initiation, and execution of apoptosis.

Although mitochondria possess their own genome, they are semi-autonomous because most of their proteins are encoded by the nuclear genome. The mtDNA is a 16.5kpb double-stranded, circular-shaped DNA molecule which encodes for 13 proteins of the mitochondrial respiratory chain, 22tRNAs and 2rRNAs required for mitochondrial protein synthesis. mtDNA transcription is dependent on three factors encoded by nuclear genes: mitochondrial RNA polymerase (POLRMT), mitochondrial transcription factor B2 (TFB2M) and mitochondrial transcription factor A (TFAM). Less than 10% of the entire mtDNA is represented by the non-coding displacement (D)-loop which overall integrates nuclear-encoded events into the transcription and replication of the mtDNA. In the same cell, some mitochondria can contain mtDNA mutation, a characteristic called heteroplasmy, while some others may contain a uniformly wt- or mutated mtDNA. Unlike nuclear DNA, mtDNA lacks histones, therefore it is more susceptible to free radicals and its repair capacity is lower compared to nuclear DNA [74]. So far, several genetic alterations both in mtDNA and nuclear genome have been identified in tumor cells. As described by Singh et al., different studies have already highlighted that alterations in mtDNA can result in chemotherapy resistance [75].

However, some studies also suggested how mtDNA variations can be induced by chemotherapy [76,77,78]. Nonetheless, limited studies have examined cisplatin activity on mtDNA of cancer cells, and the molecular mechanisms involved in mtDNA-mediated drug resistance is not well understood. 

In support of the role for mitochondria dysfunction in drug resistance, several studies with cells chemically depleted of their mtDNA (rho0 cells) have been performed.

Rho0 cells generated from normal intestinal epithelial cell lines (IEC-6) showed a significantly increased resistance to CDDP treatment compared to their parental counterparts [79].

Park et al. have shown that hepatocarcinoma cells depleted from mtDNA were less sensitive to ROS-inducing agents including doxorubicin, sorafenib, and CDDP [80]. This evidence is also associated with an activation of the nuclear factor erythroid2(NF-E2)-related factor2(NRF-2) signaling pathway and upregulation of Multidrug Resistance-associated Protein1(MRP1) and 2 (MRP2) [81]. Montopoli et al. (2011) also have demonstrated that in rho0 clones derived from ovarian cancer cells 2008 (wt) and C13 (the correspondent CDDP-resistant clone), the potency of CDDP was significantly reduced in 2008-rho0 but not in C13-rho0 when compared to their parental line [82]. This suggests that mtDNA is a target of CDDP and also that mitochondria are pivotal in the apoptotic process [82]. 

Recent studies underline how also alterations on mtDNA copy number are implicated in resistance phenomena. Mei et al. (2015) showed that lowering the copy number of mtDNA sensitizes cells to cisplatin. Their studies suggest that mtDNA copy number variation might be a novel therapeutic target for clinical treatment of tumors; in fact, their results showed that the increase of mtDNA copy number is a self-protective mechanism of tumor cells to prevent apoptosis. The reduction of mtDNA copy number by transfection with shRNA-TFAM plasmids or treatment with ethidium bromide significantly increases ROS levels and the sensitivity to cisplatin and doxorubicin [83].

In the light of these results, it seems to be some clear evidence that mtDNA alterations could concur to resistance to chemotherapy and that some drugs, cisplatin included, directly interact with mtDNA. Targeting mtDNA could lead to novel therapies for aggressive cancers; however, it is essential to underline that the fundamental step to attack mtDNA by cisplatin requires an optimal delivery system able to reach the mitochondrial matrix where the mtDNA is located [84]. 

## 7. Mitochondrial Dynamics

The focus of interest of this review is the involvement of mitochondria in cisplatin resistance and mitochondrial dynamics has been shown to assume an important role also in resistance of tumor cells to chemotherapeutic drugs such as cisplatin. 

To survive to internal and external stressors, cells need to maintain a balance of the key intracellular parameters to restore homeostasis. One of the main actors in this process is the mitochondrion. In fact, mitochondrial responses to stress are central to cell maintenance and fate. The mechanisms of mitochondrial adaptation to various stressors, including chemotherapeutic drugs, involve a reshape provided by mitochondrial motility, mitochondria fusion and fission processes and other homotypic/heterotypic interactions (such as tethering with the endoplasmic reticulum) [85,86].

Investigating the interplay between mitochondrial dynamic responses and stressors is essential to understand the shift from health to disease, as well as from sensitive to drug-resistant phenotype. Mitochondrial functions and their kind of stress response are strictly linked with their structure. These organelles have drastically different morphologies and shapes depending on the cell type and, even in the same cell, can present different morphologies [85,86]. 

In the 1980s live cell microscopy studies showed that mitochondria are organized in a fluidly interconnected, dynamic network, and their morphology and locations are not fixed but can vary depending on cell type, physiological content, degree of development, etc. [87]. The dynamic behavior of the network is essential for distribution, remodeling, and coordination of cell death programs. Moreover, it allows the remodeling necessary to respond to different stressors [88,89,90]. The multitudes of different functions of mitochondria are reflected in their structure: they present an outer and an inner mitochondrial membrane (OMM and IMM, respectively), which border the intermembrane space (IMS) and the matrix. The IMM and the OMM associate at contact points forming inward folding named cristae which accommodate respiratory chain complexes [91].

In recent years, research on “mitochondrial dynamics”, a term which includes mitochondrial fusion and fission processes, gained much attention; in fact, the balance between these two opposite processes regulates mitochondrial abundance, size, and distribution within the cytoplasm, and allows compensatory changes when cells are challenged [92,93,94,95,96].

Mitochondria fusion is the union of two mitochondria resulting in one mitochondrion; the organelle movements along cellular tracks permit the encounter between two different mitochondria facilitating the fusion process. Fusion helps cells to mitigate stress by sharing multiple elements which sustain mitochondrial biology as a form of complementation [97]. The fusion process is regulated by large guanosine triphosphatases (GTPases) of the dynamin family. The fusion of the outer membrane is mediated by membrane-anchored dynamin family members named MFN1 and MFN2 in mammals, whereas fusion between inner membranes is mediated by single dynamin family member called OPA-1 (Optic Atrophy type 1) in mammals [98,99,100,101]. On the opposite side, mitochondrial fission is characterized by the division of one mitochondrion in two daughters; this process is required for segregation of damaged mitochondria for mitophagy, for mtDNA replication and mitochondria redistribution and motility during cell division [101,102,103]. The main proteins involved in the process of mitochondrial fission are the dynamin-related protein 1 or DRP1 and fission protein homolog 1 or FIS1 which, during the fission process, localize at the level of the OMM [104].

The role of imbalance of mitochondrial dynamic in several types of cancer is established and well documented in multiple works. These studies demonstrated that increased fission activity and/or decreased fusion which lead to a fragmented mitochondrial network, occur in cancer cells [29,104,105,106,107,108,109].

A comparison between the percentage of cells with tubular mitochondria in gynecological and breast cancer cells sensitive and resistant to chemotherapy, showed a higher level of mitochondrial fusion processes in resistant cells compared to the sensitive ones [110]. This suggests that the mitochondria fusion can promote cell survival, due to a better mitochondrial activity, through an efficient production of ATP and its transport. 

Conversely, Catanzaro et al. (2015) showed that resistant ovarian cancer cells C13 present a more fragmented mitochondrial network compared with the sensitive clone 2008 [111]. 

Additionally, the inhibition of the fragmentation process has been shown to reduce the release of cytochrome c delaying cell death. Fang et al. (2012) reported that lung adenocarcinoma cells with an overexpression of OPA-1 are more resistant to cisplatin treatment [112]. OPA-1 downregulation has been shown to increase the mitochondrial cristae deformation with possible correlation to an increased release of cytochrome c, inducing apoptosis [113]. Accordingly, silencing the expression of OPA-1 may decrease the cisplatin resistance. OPA-1 -mediated mitochondrial fusion is potentially responsible for cisplatin acquired resistance in neuroblastoma B50 rat cells as demonstrated by Santin et al. (2013) [114]. Moreover, the inhibition of mitochondrial fusion by silencing of MFN1 increases cisplatin sensitivity in human neuroblastoma cells [115]. Han et al. (2017) have demonstrated that in L1210 cells subjected to cisplatin stress, MFN1 and MFN2 were upregulated, suggesting that the upregulation of mitofusins might be a cause which concurs in the development of cisplatin resistance [116].

Different studies have reported a relationship between DRP-1 regulation of fission and chemoresistance in gynecological cancer. A study of Farrand et al. (2013) demonstrated that piceatannol enhances cisplatin sensitivity in OVCA inducing the dephosphorylation of serine 637 of the DRP1, thus promoting mitochondrial fission and apoptosis [117]. Nevertheless, a different study underlined how a downregulation of DRP1 leads to drug sensitivity in ovarian cancer cells [118]. 

An overexpression of DRP-1 has been shown to lead to cisplatin resistance in lung cancer [48]. Therefore, DRP1 can play a pivotal role in conferring sensitivity or resistance to cisplatin.

## 8. Mitophagy and Chemoresistance

Autophagy is an evolutionary conserved cellular process, that plays a central role in the maintenance of cellular balance and physiology. Through the autophagic machinery damaged organelles and dysfunctional proteins are degraded allowing cells to maintain their homeostasis [119].The role of autophagy in cancer is controversial and depends on cancer type, genetic context, stage of disease, etc. [120]; nevertheless, it is well documented that the onset of autophagy allows cancer cells to elude cell death by apoptosis induced by chemotherapeutic agents but controversial results were obtained regarding chemoendocrine therapy and inhibitors of autophagy [121,122,123]. 

Cisplatin-resistant ovarian cancer cells have been shown to present elevated autophagic flux [124,125] and different studies have shown how different autophagy inhibitors can increase the sensitivity of hepatocarcinoma cells to cisplatin [126,127]. It was previously reported that cisplatin decreases mitochondrial membrane potential inducing a reduction in ATP content and an increase in ROS species. Timely removal of damaged mitochondria is thus critical for cellular homeostasis. [128]

Mitophagy (mitochondria-specific autophagy) is an essential process which contributes to mitochondrial quality control and maintenance of normal cellular physiology [129,130,131]. As a selective form of autophagy, mitophagy exploits the same core machinery of autophagy for the formation of autophagosomes and autolysosomes. However, this process needs a different priming process to label the designed mitochondria and different mitophagy effectors have been identified (including the PINK1/Parkin pathway and the mitophagy receptors NIX, BNIP3, and FUDNC1) [132,133]. The crosstalk between autophagy/mitophagy, apoptosis and mitochondrial dynamics seems to be critical for cell response to cell death induction, thus having high relevance in therapy [134]. In fact, as mitophagy represents a pro-survival mechanism in cell metabolism, its suppression in cancer cells can facilitate the elimination of malignant cells. Several evidences have shown that mitochondrial fission is a pre-requisite for mitochondrial-specific autophagy in mammalian cells. The fission process, triggered by DRP1, was found to be necessary for the segregation of damaged organelles for elimination via mitophagy.

Suppression of DRP1 by siRNA has been shown to block cisplatin-induced mitochondrial fission, mitochondrial dysfunction, and mitophagy. A study of Zaho et al. (2017) showed that inhibition of DRP1 can ameliorate cisplatin nephrotoxicity. They demonstrated that cisplatin treatment induces mitophagy which was enhanced by autophagy activators and suppressed by autophagy inhibitors [135]. Moreover, liensinine, an inhibitor of mitophagy, sensitized breast cancer cells to chemotherapy [136]; Chen et al. (2017) have demonstrated that galectin-1 overexpression, triggering autophagy, can lead to chemoresistance to cisplatin in epithelial ovarian cancer [137]. Similar results have been observed in hepatoma cells, where galectin-1-triggered autophagy seems to help cells to resist to chemotherapy drugs by eliminating dysfunctional mitochondria [138]. In fact, galectin-1 treatment is able to induce an upregulation of BNIP3 and a down regulation of m-TOR in hepatoma cells.

Different studies revealed how genetic inhibition of mitophagy pathways sensitizes cancer cells to anticancer treatment. Downregulation of mitophagic receptors like PINK1, FUNDC1 or AMBRA1 chemosensitizes cancer cells [139,140].

It has been shown that stimulation of mitophagy suppressed cisplatin-induced apoptosis in HCT116 (B) and SK-N-BE cells while inhibition of mitophagy stimulates apoptosis and autophagy [141]. In fact, the suppression of mitophagy leads to an overproduction of ROS and the fate of cells was shown to be dependent on the interplay between ER stress and autophagy.

From these recent studies it turns out that an efficient mitophagy could be a key mechanism which leads to the failure of activation of the apoptotic pathway inducing an increase in resistance of cells to chemotherapeutic treatments [142]. A better understanding of the molecular mechanisms involved in cancer resistance could provide the basis for a new approach to develop autophagy/mitophagy modulators as targeted therapies.

## 9. Conclusions

The development of novel pharmacological approaches and new pharmacological targets becomes a high priority in chemotherapy. The large amount of information generated at the mitochondrial level is expected to expand the knowledge of cisplatin resistance, and hopefully could lead to the identification of possible biomarkers for an early prediction of cisplatin therapy response. Once the specific mechanisms involved in platinum activity/toxicity at mitochondrial molecular level and cell resistance have been elucidated, inhibitors of those pathways could be developed for a synergic combination with cisplatin. The delivery of Pt-based drugs to mitochondrion together with suitable inhibitors of the antioxidant cellular system—which is enhanced in cisplatin-resistant cancer cells—can promote alternative pathways for Pt-based drugs activity. This opens the novel perspective of potential therapeutic strategies aiming at re-establishment of the response to platinum-based chemotherapy. In literature, there are recent works that support these hypotheses, presenting potential and promising approaches even if so far only in vitro studies are present. As an example, Wandee et al. (2019) showed the chemosensitizing activity of metformin in combination with cisplatin in cholangiocarcinoma. This effect is associated with an increase in oxidative stress related to mitochondrial dysfunction and initiation of cell death [143]. Additionally, melatonin sensitizes head and neck squamous carcinoma cell lines to cisplatin by the increase of mitochondrial function and following ROS overproduction [144].

Even if the findings presented in this review highlight the central role of mitochondria in cisplatin resistance, the specific mechanisms affecting mitochondrial functions still remain to be further elucidated. Study of the interaction between cisplatin and mitochondria, as well the morphological alteration that it involves, might open up new perspectives in the identification of novel mitochondrial-selective targeting strategies able to ameliorate cisplatin efficacy.

**Figure 1:**
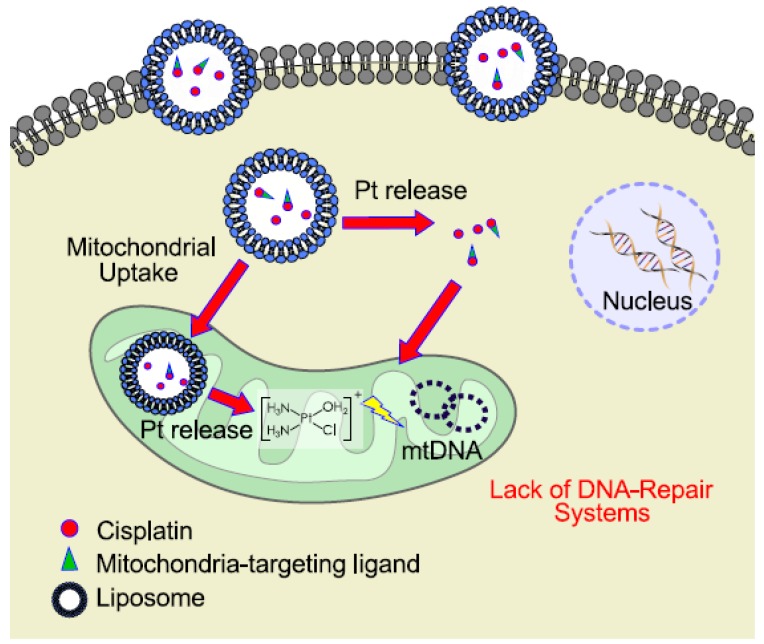
Schematic representation of an example for mitochondrial delivery of cisplatin prodrug using liposomal formulation.

With the aim of targeting directly cisplatin to mitochondria, specific drug delivery systems have been proposed and are still in development. The targeted delivery to mitochondria could limit systemic toxicity and reduce the onset of resistance phenomena. In this context, nanoparticles hold great promise in drastically change the face of oncology, thanks to their ability of targeted delivery avoiding undesirable biodistribution, severe systemic effect, and drug resistance. In this field, Marrache et al. (2014) developed a mitochondria-targeted Pt(IV)-prodrug of cisplatin delivered in a biocompatible polymeric nanoparticle (NP); this formulation allows the delivery of platinum inside the mitochondria of neuroblastoma cells resulting in 17 times more activity than cisplatin [84]. Notably, the exploitation of delivery strategies to improve the antitumor activity of cisplatin has recently attracted considerable attention since they can offer significant tools to ameliorate the drug efficacy and reduce toxicity and side effects as shown in Figure 1 [7,145,146,147]. Among the delivery strategies that are considered more promising for the clinical translation, liposomal loaded cisplatin (Lipoplatin) has advanced to Phase III human clinical trial, showing superiority to cisplatin as a chemotherapy regimen in non-small cell lung cancer (NSCLC) adenocarcinomas.

Even though the mitochondrion appears as a tempting target for anti-cancer therapies, the huge disparity in the reported mitochondrial alterations among tumors could interfere with the efficacy of the potential mitochondrial-targeted treatments. Another highly significant result emerged from clinical applications of mitochondrial-targeted drugs in cancer therapies is that they have shown limited side-effects on normal “healthy” cell populations in vivo [148]. However, it is still too early to judge the clinical impact that mitochondrial-targeted drugs will make in treating cancer.
